# Direct Intra-articular Antibiotic Administration for Acute Prosthetic Joint Infection in Knee Arthroplasty

**DOI:** 10.7759/cureus.26612

**Published:** 2022-07-06

**Authors:** Wan Lye Cheong, Yi Xiang Tan, Teck Siong Fong, Mohamed Nazri Mohamed Nazeeb, Tuck Shin Fong

**Affiliations:** 1 Orthopaedic Surgery, Putrajaya Hospital, Putrajaya, MYS; 2 Orthopaedic Surgery, Columbia Asia Hospital Bukit Rimau, Shah Alam, MYS

**Keywords:** biofilm, debridement and implant retention, total knee arthroplasty, prosthetic joint infection, intra-articular antibiotic

## Abstract

Prosthetic joint infection (PJI) remains a challenge to treat. We utilized intra-articular administration of antibiotics for the treatment of two infected total knee arthroplasties. The first patient developed an early post-operative infection with persistent wound drainage within a week after primary total knee arthroplasty (TKA). The second patient had an acute hematogenous infection, presenting with knee pain with a preceding history of leg cellulitis, one year after a primary TKA. Both patients were treated with surgical debridement, exchange of tibial insert with implant retention, and intra-articular administration of vancomycin for six weeks. Treatment was successful for both patients, with preservation of knee function and no recurrence of infection after one year. We reported two cases of PJI treated with direct intra-articular antibiotic administration following surgical debridement and implant retention.

## Introduction

Prosthetic joint infection (PJI) is a dreaded complication of total knee arthroplasty (TKA). Its incidence ranges from 1% to 2% and carries a significant burden on patients and healthcare systems, including prolonged hospitalization, multiple surgeries, prolonged use of antibiotics, and extended duration of functional impairment [1]. The estimated treatment cost for revision surgery may be three to four times compared to a primary TKA [2]. Multiple treatment strategies exist to treat PJI, depending upon factors such as the chronicity of infection, soft tissue condition, bone stock, and identification of organism pre-operatively [1]. Traditionally, a two-stage exchange arthroplasty is considered the gold standard treatment for PJIs. However, due to its associated morbidity, multiple modalities have been explored. Single-stage revision or debridement, antibiotics and implant retention (DAIR) methods are increasingly used for acute PJIs, but the success rate varies [3]. To improve the outcome of these methods, intra-articular antibiotic administration has been employed, with a reported success rate as high as 95% [4].

## Case presentation

Case A

Patient A is a 74-year-old lady with underlying hypertension and lumbar spondylosis. She underwent right total knee arthroplasty for degenerative osteoarthritis. One week postoperatively, there was persistent haemoserous drainage from the wound site. There was no erythema, range of motion of the knee was 0 to 90 degrees. She was afebrile. Inflammatory markers were as follows: white blood cell (WBC) count of 7.0x10^9^/L, C-reactive protein (CRP) of 128 mg/L, erythrocyte sedimentation rate (ESR) of 71 mm/h (Table 1). She was started on intravenous (IV) vancomycin 1g 12-hourly. A prior joint fluid culture was negative. However, one week later, the wound discharge persisted. A Synovasure® test (Zimmer Inc., Warsaw, IN, USA) of her knee joint fluid aspirate was then performed and returned positive, indicating infection by detecting levels of alpha-defensin. She underwent an aggressive debridement, excision of necrotic and fibrous tissue, and inflamed synovium, with the exchange of tibial insert and retention of an implant. Intra-operatively, there was serous fluid and sloughy tissue. There was no loosening of the tibial and femoral components, and no biofilm was observed. Six specimens (tissue specimens from suprapatellar, infrapatellar, medial and lateral gutter, femur intercondylar notch, and bone) were sent for culture, all of which grew *Enterococcus sp.*, with susceptibility towards vancomycin. Two Jet-Cath® (Jet Medical, SA, Switzerland) catheters were placed. These catheters serve as portals for intra-articular (IA) administration of vancomycin.

**Table 1:** Biochemical parameters and organism cultures for cases A and B *initial presentation in another hospital **presentation one week after IV antibiotic IA - intra-articular

	Case A	Case A (after IA antibiotic treatment)	Case B*	Case B**	Case B (after IA antibiotic treatment)
White blood cell count (WBC)	7.0x10^9^/L	5.9x10^9^/L	21.4x10^9^/L	15.4x10^9^/L	9.0x10^9^/L
C-reactive protein (CRP)	128 mg/L	<5.0 mg/L	62.6 mg/L	92.0 mg/L	<5.0 mg/L
Erythrocyte sedimentation rate (ESR)	71 mm/h	26 mm/h	61 mm/h	96 mm/h	30 mm/h
Synovial white blood cell count	NA	NA	62x10^3^ cells/µL	NA	NA
Organism culture	Enterococcus sp.	NA	Streptococcus dysgalactiae	NA	NA

Case B

Patient B is a 65-year-old male with underlying hypertension and a history of bilateral TKA; right and left TKA was performed eight and one year prior, respectively. The patient was well following the primary TKA up until the current onset of symptoms. He presented to another institution initially with fever, left knee pain, and breathlessness. This was preceded by ipsilateral leg cellulitis of one-week duration. Serum inflammatory markers were high: WBC count of 21.4x10^9^/L, ESR of 61 mm/h, CRP of 62.6 mg/L. Left knee aspiration yielded cloudy fluid, a raised synovial WBC count of 62x10^3^cells/µL with 83% neutrophil, and *Streptococcus dysgalactiae* was isolated (Table 1). Intravenous (IV) vancomycin and piperacillin-tazobactam were started, and he was subsequently referred to our center for further treatment. Upon our review one week after the initial presentation, his left knee was warm and tender, with limited range of motion. Laboratory data revealed a serum WBC count of 15.4x10^9^/L, ESR of 96 mm/h, and CRP of 92.0 mg/L (Table 1). A plain radiograph of his left knee did not show signs of osteolysis, periosteal reaction, lucency, or loosening of the implant. Intravenous vancomycin 1g 12-hourly was continued, and the patient underwent surgical debridement and a change of tibial insert. Intra-operatively, there was seropurulent fluid within the knee joint and sloughy tissue. There was no loosening of the tibial and femoral components, and no biofilm was observed. Six peri-prosthetic tissue specimens (tissue specimens from the suprapatellar, infrapatellar, medial and lateral gutter, femur intercondylar notch, and bone) were sent for culture, and all results were negative. Intra-operatively, two Jet-Cath® catheters were inserted. The intra-operative cultures were likely negative as antibiotic therapy had already been initiated for a week prior to presentation at our center. He was treated as PJI in view of clinical signs of inflammation, elevated biochemical and synovial parameters, purulent synovial fluid, and positive culture. Vancomycin was selected as the antibiotic choice in accordance with the culture result from the referring institution.

Catheter Insertion Technique

**Figure 1:**
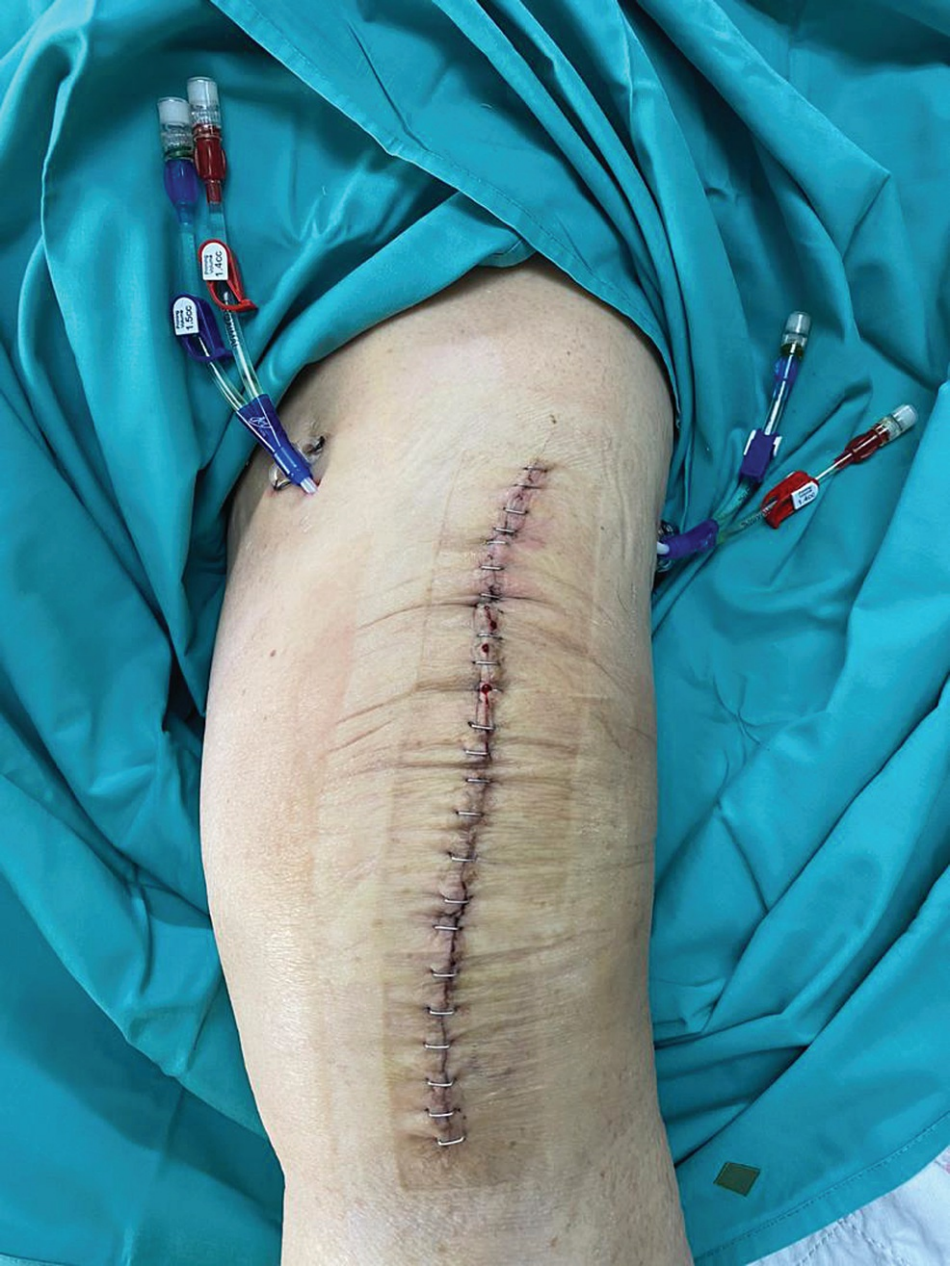
Placement of two Jet-Cath® catheters on the superomedial and superolateral side of the left knee Case B: these catheters serve as portals for intra-articular administration of antibiotics.

Two Jet-Cath® catheters were inserted percutaneously at the superolateral and superomedial aspects of the knee prior to wound closure (Figure 1). These are double-lumen catheters frequently used for venous access for hemodialysis. A small stab incision was made, and a track was created via blunt dissection with straight artery forceps. The provided guide wire was tunneled through the vastus lateralis or medialis muscle into the knee joint under direct vision. The catheter was then threaded along the guide wire, its tip positioned within the joint, and the guide wire removed. A single stitch was performed near the catheter entry point to ensure a snug fit. The catheter was subsequently anchored to the skin with a non-absorbable suture. Powdered vancomycin 500 mg diluted in 5 mL of water was used to prime the catheter. Betadine® ointment was applied over the catheter entry site and around the Luer lock port prior to securing with the included caps. A keyhole gauze and sterile transparent dressing were then applied.

Intra-articular Antibiotic Regime

**Figure 2:**
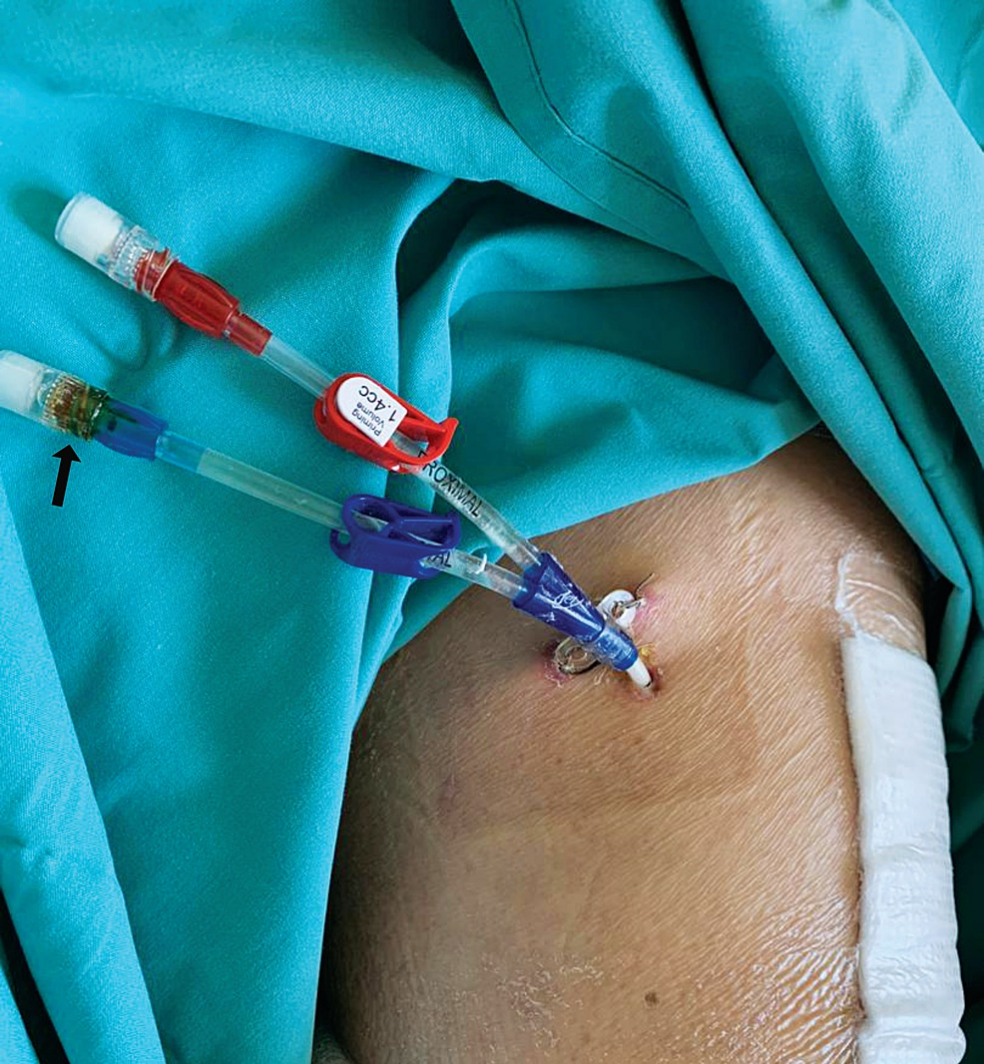
Intra-articular catheter over the medial aspect Case B: Betadine® ointment (black arrow) was used to seal the injection port of the blue catheter.

Administration of vancomycin through the catheter was performed under an aseptic technique. IA vancomycin was given through the lateral side catheter for easier access and care of the portal site. In the event of catheter blockage or dislodgement, IA vancomycin delivery can be switched to the medial catheter. Betadine® ointment was used to seal the port after each administration of an intra-articular antibiotic (Figure 2). Care was taken to apply the ointment surrounding the port thread only so that the lumen was not occluded. The catheters were never flushed with any solution following the antibiotic injection to ensure patency and reduce contamination. The portal site was wrapped with sterile gauze and sealed with a waterproof, transparent film dressing to avoid soiling.

**Figure 3:**
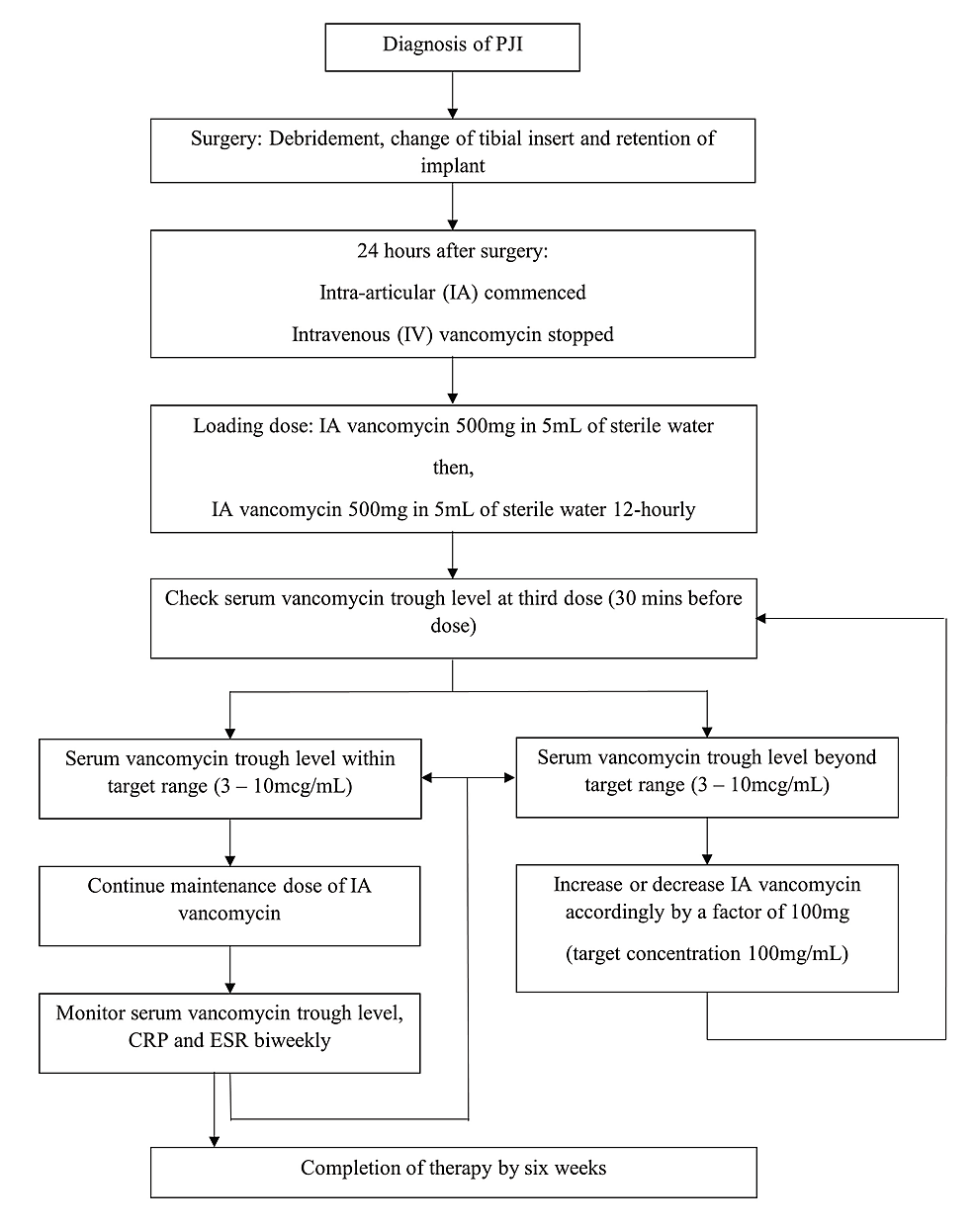
Protocol for intra-articular antibiotic (vancomycin) administration PJI - prosthetic joint infection; CRP - C-reactive protein; ESR - erythrocyte sedimentation rate

For both patients, IA vancomycin was commenced, and IV vancomycin was stopped on day one postoperatively. Our protocol for IA vancomycin administration was, as shown in Figure 3, adapted from Whiteside et al. [4]. IA vancomycin 500 mg 12-hourly was given and adjusted accordingly to maintain a serum vancomycin trough level of ­3­-10 mcg/mL. Serum vancomycin trough level, CRP, and ESR were monitored bi-weekly. After six weeks, the IA antibiotic was completed, and the catheters were removed. In case B, the lateral catheter entry site developed a local skin reaction, necessitating early removal and use of the medial catheter for the continuation of the IA vancomycin. The infection resolved in both knees, both clinically and biochemically. At three, six, and twelve months postoperatively, both knees showed no evidence of recurrence of infection and maintained good knee function. Both patients were able to walk independently, without pain over the affected knee, and had a good range of motion (knee extension 0 degrees, flexion 100 to 140 degrees).

## Discussion

Treatment of prosthetic joint infection is complex and challenging. The gold standard treatment of PJI is two-stage exchange arthroplasty. Following Tsukayama's classification of PJI and the Infectious Diseases Society of America guideline, early postoperative (within four weeks of surgery) and acute hematogenous infections (symptoms onset within three weeks) can be considered candidates for DAIR, thereby reducing the morbidity of a two-stage procedure [1,5]. However, the success rate remained variable in the literature. The success rate of DAIR has been reported to be between 31 to 78%. Risk factors for DAIR failure include virulence of organisms and retention of modular components [3]. This highlights the important concept of biofilm formation, produced especially by more virulent organisms such as *Staphylococcus aureus*. Biofilms reduce antimicrobials penetration and provide a matrix for the growth of microorganisms which are slower growing and resistant to modest levels of antimicrobials [6]. A high concentration of antimicrobial will be more effective against the resistant microorganisms, and, when sustained, will allow more time for antimicrobial penetration and action on slower-growing pathogens. Conventionally, intravenous antibiotics are used for this purpose. However, the synovial concentrations achieved, although above the minimal inhibitory concentration of the organism, are often not adequately effective when biofilms are involved [7]. Biofilm pathogens require a much higher level of local antimicrobial and for a longer duration. When given intravenously, synovial antimicrobial levels would fall below the therapeutic level shortly after administration. On the other hand, a higher intravenous dose would risk systemic toxicity.

With the ability to maintain a sustained high intra-articular antimicrobial concentration effective against biofilm pathogens, an intra-articular antibiotic may reduce the need for two-stage procedures, provided debridement is adequate. Whiteside et al. reported treatment success in two cohorts of patients involving multi-resistant organisms and reinfection cases following failed revision surgery. Both groups saw 17 out of 18 patients cleared of infection after single-stage revision and intra-articular antibiotics. The patients who failed underwent another round of revision surgery and IA antibiotics and were subsequently infection-free [4]. Antony et al. treated 57 patients with intra-articular antibiotics following one- or two-stage revision and found 51 patients (89%) infection-free at 11-month follow-up. Six who failed have had prior complex procedures and infection with resistant organisms [8]. Fukagawa et al. demonstrated treatment success in 11 knees with chronic or hematogenous infection. They were treated with debridement, implant retention, and the exchange of modular components [9]. In another retrospective study, 46 out of 51 patients (90.2%) with culture-negative PJI were successfully treated with intra-articular antibiotics and one-stage revision. As the pathogens were not identified, the patients were given an empirical combination of intra-articular vancomycin and imipenem [10]. Conventionally, culture-negative PJI is treated with two-stage surgery as this allows a second attempt at obtaining tissue for culture.

Reported adverse effects of IA antibiotic treatment were local inflammatory reaction secondary to precipitated antibiotic, acute renal failure, and catheter leakage or failure. However, the rates are low, and all cases of acute renal failure are reversible, following temporary cessation of intra-articular antibiotic for not more than a few days and subsequent dose reduction. Most of the local reactions resolved with temporary discontinuation of therapy [4,8,10].

The catheters should be handled in a sterile manner to reduce the risk of contamination and infection. Local inflammatory reaction at the catheter entry site is possible, especially with leakage of an antibiotic precipitate. Properly securing the catheter and applying adequate dressing around the entry site may reduce this complication. During the course of therapy, the catheter may experience malfunction or blockage, or local skin reactions may develop. With the insertion of two catheters, one on each side, there is a spare catheter to be used in the event such complications develop. Currently, there is no clear guideline on antibiotic choice or concentration to be used. General strategy involves administration of an initial dose followed by adjustment according to therapeutic serum level. Following an adaptation of Whiteside's protocol, we found that IA vancomycin 500 mg 12-hourly were able to maintain adequate serum trough level in our patients [4]. We would suggest further studies to determine the best loading and maintenance dose, frequency of monitoring of serum level, and the target serum trough level for the safe and effective administration of intra-articular antibiotics following DAIR.

There is reported use of adjunct agents such as rifampin or fusidic acid as combination therapy in the literature. However, resistance may easily develop, and side effects may occur, as this is a systemic treatment. The success rate of combination therapy also depends on several factors. For example, rifampin combination therapy is only effective when administered in a young age biofilm, with low bacterial load and intact soft tissue conditions [11]. In addition, these studies compared only the systemic use of the combination therapy. Further studies to gauge the effectiveness of intra-articular antibiotics alone versus combination therapy would be interesting and beneficial.

## Conclusions

Intra-articular administration of antibiotics demonstrated good outcomes in our treatment of prosthetic joint infection. It serves as an adjunct to surgical treatment, whether one-stage exchange arthroplasty or debridement with implant retention, reducing morbidity often associated with a two-stage surgery. The basis of this method is the ability to maintain a sustained high local concentration of antimicrobials for the eradication of biofilm-producing microorganisms. It is most suitable to be used in early postoperative or acute hematogenous infection with identified susceptible organisms. These two cases highlight our success, and we believe further large-scale multi-center studies would be able to validate this approach to the management of PJI.
